# Amine-Functionalized Natural Rubber/Mesostructured Silica Nanocomposites for Adsorptive Removal of Clofibric Acid in Aqueous Phase

**DOI:** 10.3390/molecules28052330

**Published:** 2023-03-02

**Authors:** Satit Yousatit, Witsarut Rungruangwattanachot, Natthakit Yuwawanitchakorn, Sakdinun Nuntang, Patiparn Punyapalakul, Chawalit Ngamcharussrivichai

**Affiliations:** 1 Department of Chemical Technology, Faculty of Science, Chulalongkorn University, Bangkok 10330, Thailand; 2 Center of Excellence in Catalysis for Bioenergy and Renewable Chemicals (CBRC), Faculty of Science, Chulalongkorn University, Bangkok 10330, Thailand; 3 Industrial Chemistry Innovation Programme, Faculty of Science, Maejo University, Chiang Mai 50290, Thailand; 4 Research Unit Control of Emerging Micropollutants in Environment, Faculty of Engineering, Chulalongkorn University, Bangkok 10330, Thailand; 5 Center of Excellence on Petrochemical and Materials Technology (PETROMAT), Chulalongkorn University, Bangkok 10330, Thailand

**Keywords:** mesoporous materials, nanocomposite, adsorption, amine group, clofibric acid

## Abstract

This study is the first report on the synthesis, characterization and application of amine-functionalized mesoporous nanocomposites based on natural rubber (NR) and wormhole-like mesostructured silica (WMS). In comparison with amine-functionalized WMS (WMS-NH_2_), a series of NR/WMS-NH_2_ composites were synthesized via an in situ sol-gel method in which the organo-amine group was grafted onto the nanocomposite surface via co-condensation with 3-aminopropyltrimethoxysilane (APS) as the amine-functional group precursor. The NR/WMS-NH_2_ materials had a high specific surface area (115–492 m^2^ g^−1^) and total pore volume (0.14–1.34 cm^3^ g^−1^) with uniform wormhole-like mesoporous frameworks. The amine concentration of NR/WMS-NH_2_ (0.43–1.84 mmol g^−1^) was increased with an increase in the APS concentration, corresponding to high levels of functionalization with the amine groups of 53–84%. The H_2_O adsorption–desorption measurement revealed that NR/WMS-NH_2_ possessed higher hydrophobicity than WMS-NH_2_. The removal of clofibric acid (CFA), a xenobiotic metabolite of the lipid-lowering drug clofibrate, from the aqueous solution using WMS-NH_2_ and NR/WMS-NH_2_ materials was investigated using a batch adsorption experiment. The adsorption was a chemical process in which the pseudo-second order kinetic model expressed the sorption kinetic data better than the pseudo first-order and Ritchie-second kinetic order model. In addition, the CFA adsorption sorption equilibrium data of the NR/WMS-NH_2_ materials were fitted to the Langmuir isotherm model. The NR/WMS-NH_2_ with 5% amine loading had the highest CFA adsorption capacity (6.29 mg g^−1^).

## 1. Introduction

Pharmaceutical residues contaminated wastewater has been receiving attention from scientific and governmental organizations due to perceived threats to the environment and human health [1]. The leakage of pharmaceutical wastewater and its ingredients from the manufacturing process can enter the aquatic environment through wastewater treatments, therefore the varieties of pharmaceutical residues have already been found in surface water and ground water, which becomes a severe environmental problem owing to their toxicity, solubility, and pseudo persistence [2]. Clofibric acid (CFA), a xenobiotic metabolite of cholesterol-lowering pharmaceutical fibrate drugs, is considered a representative pharmaceutical residue with a high level of environmental risk because it is often detected in the aquatic ecosystem [3,4,5]. Moreover, CFA imposes negative effects on metabolism, causes reproductive damage, and impacts the embryonic development and behavior of aquatic organisms, creating further risks to human health [6,7,8]. Therefore, various procedures have been used for the removal of CFA from industrial wastewater, such as photochemical degradation, biological processes, coagulation/flocculation/sedimentation, and filtration [9,10,11]. Nevertheless, these processes have limited use due to their need for expensive equipment as well as their high reagent or energy consumption. 

Adsorption is one of the potentially effective and economical techniques for removing pharmaceutical residues from wastewater because of its ease of operation, low capital and operating cost, and low energy consumption [9,10,11]. Mesoporous silica materials have received a lot of attention due to their excellent properties, including high surface area and large pore volume, good hydrothermal and mechanical stability, and high adsorption capacity [12,13,14,15,16]. Particularly, the mesoporous silica materials functionalized with amine groups (–NH_2_) are a class of solid sorbents for the removal of CFA from aqueous solutions [3,17]. The previous study [3,17] demonstrated that the amine-functionalized mesoporous silicas exhibited good performance for the adsorption of CFA due to acid–base and/or ionic interaction and hydrogen bonding between the amino groups and carboxylic groups in CFA molecules. Nonetheless, the adsorption of organic molecules in aqueous solution is inefficient because water molecules are favorably adsorbed owing to the high density of residual silanol groups on the silica’s surface. The surface modification of silica materials by the grafting of hydrophobic and adsorption active sites can enhance selective adsorption for organic or inorganic pollutants [3,18]. However, the resulting mesostructured silicas suffer from a severe loss of surface area and pore volume by a non-uniform distribution of the organo-functional groups anchored within the mesoporous channels and on the mesopore mouth. 

The incorporation of hydrophobic polymer moieties into the mesostructure of silica is a facile approach to decrease the interaction of adsorbed water with the internal siliceous surface of mesopores, resulting in an enhanced affinity toward organic molecule adsorption in aqueous solution [13,14,15]. Nuntung et al. [13,16] prepared a series of mesoporous nanocomposites consisting of hydrophobic natural rubber (NR), a *cis*-1,4-polyisoprene, dispersed in the wormhole-like mesoporous silica (WMS). The nanocomposites obtained exhibited a well-ordered structure, large surface area and pore volume, and improved hydrophobicity. Although several studies have demonstrated that CFA can be removed effectively by mesoporous silica functionalized with organic amine groups, to the best of our knowledge, no report on the synthesis, characterization, and application of amine-functionalized NR/WMS nanocomposites for CFA removal from aqueous solutions exists. 

In this work, we report the preparation of novel mesoporous nanocomposites, based on NR and WMS fabrication via an in situ sol-gel method, followed by the functionalization of a WMS surface with organo-amine groups (NR/WMS-NH_2_) using 3-aminopropyltrimethoxysilane (APS) as the amine-functional group precursor. Their potential adsorbents were tested in the adsorption of CFA, as a metabolite of the cholesterol-lowering pharmaceutical drug, in aqueous solution. The physicochemical and adsorption properties of NR/WMS-NH_2_ were compared with those of aminopropyl-functionalized WMS (WMS-NH_2_) synthesized under similar conditions. The adsorption mechanism was also investigated by analyzing the adsorption kinetics, isotherms, and thermodynamics under various conditions. The overall results indicate that the NR/WMS-NH_2_ materials take advantage of both the hydrophobicity and the specific adsorptive interaction, leading to an enhanced affinity toward CFA adsorption. Our findings can provide a better understanding of the mechanism for adsorption of CFA onto adsorbents with improved hydrophobicity.

## 2. Results and Discussion

### 2.1. FTIR Spectroscopy

**Figure 1:**
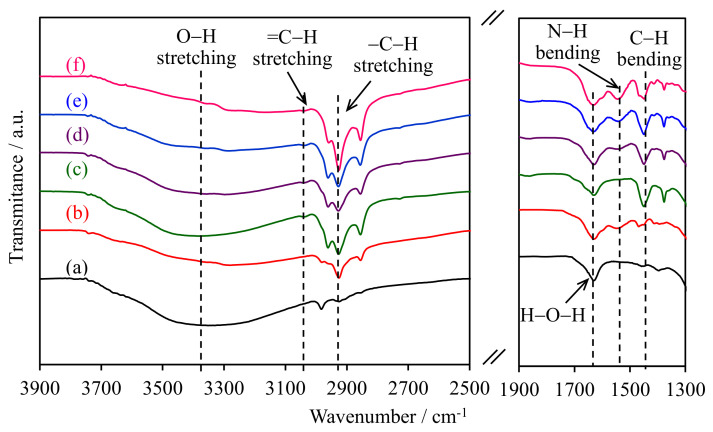
FTIR spectra of (a) WMS, (b) WMS-0.10, (c) NR/WMS, (d) NR/WMS-0.05, (e) NR/WMS-0.10, and (f) NR/WMS-0.15.

The FTIR spectra of the WMS-NH_2_ and NR/WMS-NH_2_ materials in the selected wavenumber regions are shown in Figure 1. The O–H stretching vibration of the silanol groups was observed at 3100–3700 cm^−1^ [19]. For the pure silica WMS, the band around 2930 cm^−1^ was attributed to the C–H stretching of remnant ethoxy groups as incompletely condensed silicate species [20], while the O-H bending vibration of physisorbed water was observed at 1630 cm^−1^ [21,22]. After the functionalization, the WMS-0.10N showed the bands related to the aminopropyl group at 1530 cm^−1^, corresponding to the N–H bending vibration of the amino groups, and at 2989, 2940 and 2869 cm^−1^ due to the C–H stretching of methylene moieties [22,23,24]. 

The presence of rubber entrapped in the pristine NR/WMS was deduced from an intense –C–H stretching vibration at 2989, 2940, 2869, the bands =C–H stretching at 3120 cm^−1^ and the deformation of methylene groups at 1461 cm^−1^ [15,16,25]. The bands of silanol groups and physisorbed water were less pronounced for the NR/WMS nanocomposites than the WMS counterparts, which were characterized an enhanced hydrophobicity via the incorporated rubber phase. The N–H bending band at 1530 cm^−1^ increased in its intensity with the aminopropyl content of NR/WMS-NH_2_. The result was collaborated with the Si–O–Si and Si–O–H stretching vibrations at 1000–1300 cm^−1^ and 940–960 cm^−1^, respectively [20,26,27], as shown in the Supplementary Material Figure S1. When increasing the APS loading in the NR/WMS-NH_2_, the Si–O–Si band was shifted towards lower wavenumbers (from 1065 to 1048 cm^−1^), which was associated with an increased condensation of the silicate wall [17]. On the other hand, the Si–O–H band of the non-condensed silanol groups gradually decreased. These results confirmed the successful functionalization of the NR/WMS surface with the aminosilane at different loading levels of aminopropyl groups.

### 2.2. Solid State ^29^Si CP/MAS NMR Spectroscopy

**Figure 2:**
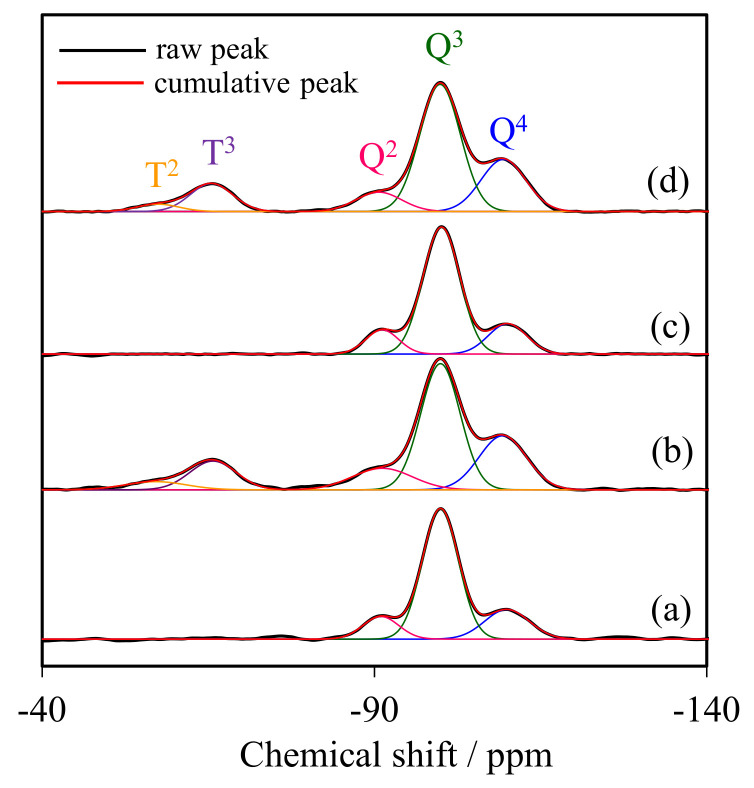
^29^Si MAS NMR spectra of (**a**) WMS, (**b**) WMS-0.10, (**c**) NR/WMS, and (**d**) NR/WMS-0.05.

Figure 2 compares the ^29^Si CP/MAS-NMR spectra of representative NR/WMS-NH_2_ with that of WMS-NH_2_ to determine the degree of amino groups anchored onto the silica surface. All samples displayed three distinct signals in the range of chemical shifts between −80 and −120 ppm, corresponding to different framework silica species (Q^n^). The signals at −92, −101 and −111 ppm were, respectively, assigned to Q^2^ (Si(OSi)_2_(OH)_2_), Q^3^ (Si(OSi)_3_(OH)) and Q^4^ (Si(OSi)_4_). The functionalization with APS gave two more signals at −57 and −67 ppm, relating to T^2^ (R–Si(OSi)_2_(OH)) and T^3^ (R–Si(OSi)_3_) species, respectively, where R represents the aminopropyl groups [28,29]. This result indicated that the amino-functional moieties were incorporated as part of the silica framework. The relative population of silicon environments was calculated by deconvolution of the NMR spectra into individual Gaussian peaks. The NR/WMS composite gave higher relative intensities of Q^2^ and Q^3^ than pure silica WMS (Table 1), indicating a high amount of free silanol and remnant ethoxy groups on the surface of WMS in the presence of NR. This result suggested that the NR molecule incorporated into the mesostructure resulted in a reduction in the hydrolysis and condensation of TEOS [13]. The fraction of T^m^ species, reported as a ƩT^m^/Ʃ(T^m^ + Q^n^) ratio, of WMS-NH_2_ (17.53%) was higher than that of NR/WMS-NH_2_ (15.09%), which was in accordance with their nitrogen content, obtained from the elemental analysis (Section 2.3). Although the presence of the rubber phase hampered the surface functionalization, a significant decrease in the relative amount of Q^2^ and Q^3^ signals of the NR/WMS-NH_2_ signified the substitution of surface silanol groups by the incorporated aminopropyl groups. 

### 2.3. Elemental Analysis

The total amount of nitrogen (mmol_N_ g^−1^) was measured by CHNS/O analysis to quantify the amino-functional groups presenting in the WMS-NH_2_ and NR/WMS-NH_2_. The pure silica WMS and NR/WMS exhibited no significant nitrogen content, confirming the successful removal of the DDA template (Table 2). The amine concentration of the NR/WMS-NH_2_ series increased with the aminosilane loading level. The NR/WMS-0.15 prepared with 15 mol% APS loading possessed the highest amine content (1.84 mmol_N_ g^−1^). In addition, increasing the APS/TEOS molar ratio of the synthesis mixture from 5% to 15% resulted in an increased yield of the aminopropyl group from 53% to 83%. Compared to the theoretical amine concentration, the degree of surface functionalization with APS was estimated to be 96% and 80% for WMS-0.10 and NR/WMS-0.10, respectively. The smaller functionalization level observed for the NR/WMS-NH_2_ nanocomposite suggested that some rubber fraction hindered the accessibility of APS to the silicate species. 

### 2.4. Thermogravimetric Analysis

**Figure 3:**
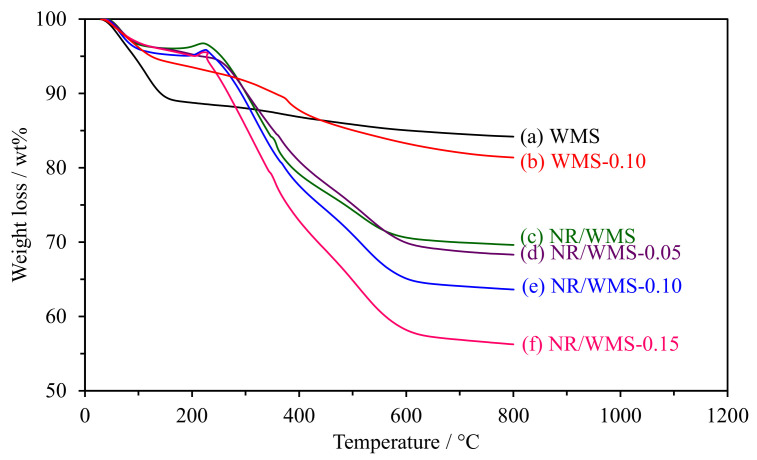
Weight loss curves of the WMS and NR/WMS prepared with different amine loadings.

The weight loss curves of WMS and NR/WMS functionalized at the different loading of the amine group are shown in Figure 3. The weight loss of pure silica WMS had no significant change in the region of 200–600 °C, indicating that the removal of the DDA template was successful after the extraction process. The weight loss below 150 °C was ascribed to a loss of physisorbed moisture and residual ethoxy groups owing to the incomplete hydrolysis of TEOS [30]. WMS-0.10N exhibited the decomposition of aminopropyl groups (~7 wt%) at 160–450 °C, which occurred in two successive steps [21,23]. The first step was found in the temperature range of 160–300 °C, corresponding to the decomposition of amino moieties, while the hydrocarbon chain of the aminopropyl groups was decomposed in the latter step (300–450 °C). A small weight loss (1–3 wt%) was observed at 500–650 °C, which was attributed to the carbon residue and dehydroxylation of the silicate network [19]. 

For the NR/WMS-NH_2_ series, the weight gain at 170–230 °C was related to the thermal oxidation of NR incorporated into the nanocomposite materials (~0.6 wt%) [31,32]. The decomposition of the hydrocarbon chain of aminopropyl groups and rubber molecules (17–20 wt%) occurred in a similar temperature range (230–450 °C) [16,21]. Compared to the pristine NR/WMS, the weight loss in this step was systematically increased with the amine loading level, which indicated the presence of amino-functional groups and rubber in the NR/WMS-NH_2_ materials.

### 2.5. XRD Analysis

**Figure 4:**
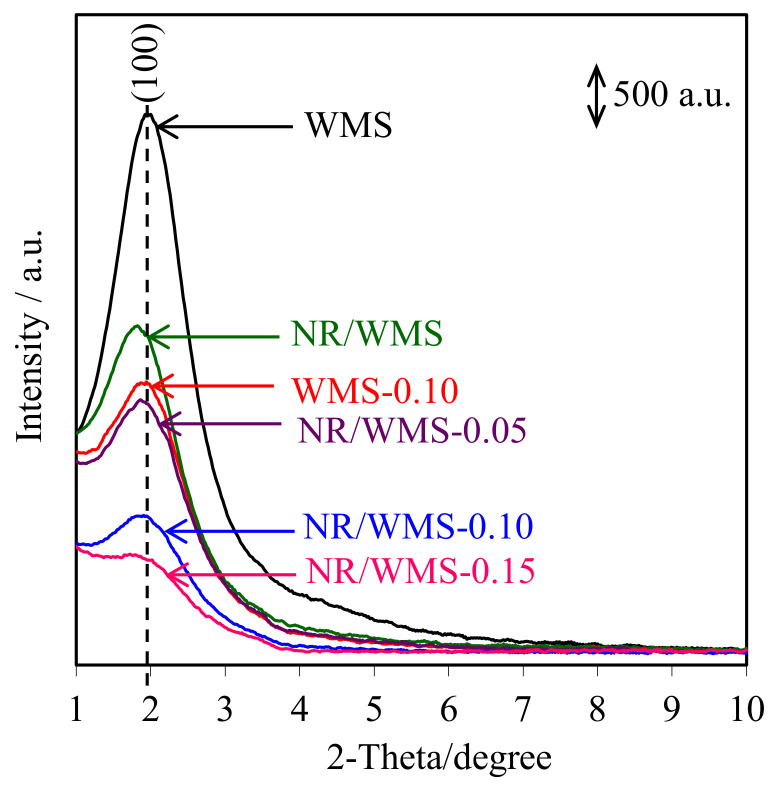
XRD patterns of the WMS and NR/WMS functionalized with different amine loadings.

The XRD patterns of pure silica WMS and NR/WMS nanocomposites prepared without and with the functionalization of an aminopropyl group are shown in Figure 4. All samples clearly exhibited a single reflection at 2*θ* around 1.5°–3.0°, corresponding to the (100) plane of wormhole-like mesostructured silicate framework [33]. The introduction of the organic species, either the aminopropyl group or NR, into the WMS decreased the structural arrangement [3,17]. The peak intensity of the NR/WMS-NH_2_ samples was lower than that of the pristine NR/WMS and decreased with increasing the APS loading level. This result can be explained by either a lower diffraction contrast or a loss of structural order when the amino-functional groups were incorporated into the mesostructured silica. Interestingly, the characteristic peak was still retained even at 15 mol% of APS loading onto the NR/WMS. Some structural data obtained from the XRD analysis are summarized in Table 2. The WMS-NH_2_ gave higher *d*_100_ and *a*_0_ than the WMS due to the expansion of pore size (see Section 2.6). An increase in the pore wall thickness (*W*_t_) after functionalization indicates that the amino-functional groups were anchored on the inner surface of the mesoporous silica [34].

Compared to the pure silica WMS, the *a*_0_ and *W*_t_ values were expanded in the case of the NR/WMS (Table 2). These results indicated not only that the rubber molecules were trapped in the mesostructured silica framework [13,16], but also the presence of the less condensed silicate framework of the NR/WMS nanocomposite. The amine-functionalization onto the NR/WMS induced the contraction of *a*_0_, while enhancing an expansion of *W*_t_. This result indicates that the amine group promoted the hydrolysis and condensation of TEOS, resulting in a thicker silicate wall. It can be rationalized from these results that the mesoporous structure of both the WMS-NH_2_ and NR/WMS-NH_2_ was functionalized with the aminopropyl group.

### 2.6. N_2_ Adsorption–Desorption Measurement

**Figure 5:**
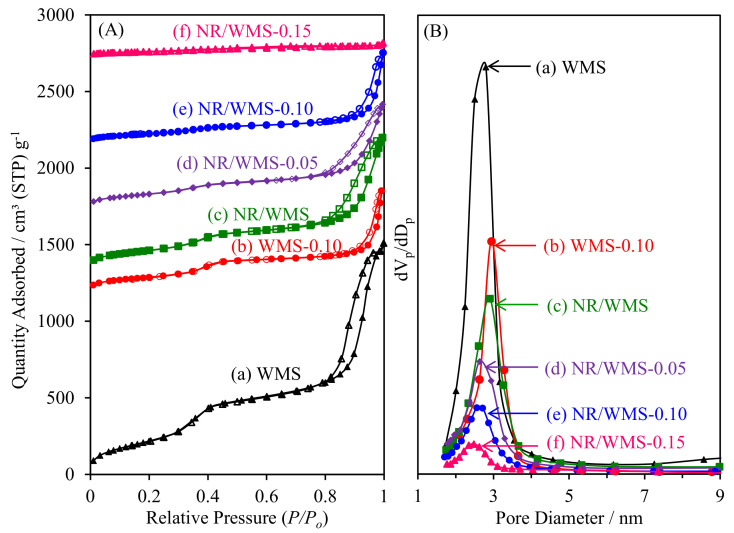
(**A**) N_2_ adsorption–desorption isotherms and (**B**) BJH pore size distribution of (a) WMS, (b) WMS-0.10, (c) NR/WMS, (d) NR/WMS-0.05, (e) NR/WMS-0.10 and (f) NR/WMS-0.15.

As shown in Figure 5A, the N_2_ physisorption isotherms of pristine and amino-functionalized WMS and NR/WMS materials were classified as type IV with a hysteresis loop according to the IUPAC classification, which are characteristics of framework confined mesoporous materials [16]. The large hysteresis loop observed at *P/P*_0_ > 0.8 originated from N_2_ condensation inside the interparticle voids of particle agglomerates. Table 2 summarizes the textural properties of the materials. The functionalized WMS and NR/WMS series exhibited lower *S*_BET_, *V*_p_ and *V*_t_, but with an increased *W*_t_, than the corresponding pristine ones due to the chemical grafting of the organo-functional group onto the mesoporous silica surface. With increasing APS loading onto the NR/WMS, the change was systematically extended to a higher degree. A decrease in *S*_ext_ after the functionalization suggested that the particle size of the WMS-NH_2_ and NR/WMS-NH_2_ was larger than that of the WMS and NR/WMS, respectively, which was explained by a catalytic effect of the basic amino group on the hydrolysis and condensation of the silicate species [17,35]. 

A major difference in the effect of functionalization on the textural properties of the WMS and NR/WMS materials was observed when taking to account the BJH pore size distribution (Figure 5B). Compared to the corresponding pristine materials, *D*_p_ was increased for WMS-NH_2_ but reduced in the case of NR/WMS-NH_2_. During WMS-NH_2_ synthesis, the presence of a hydrogen bonding interaction between the amino groups existing in the aminosilane, and those of the head groups of the DDA molecules, possibly expanded the confined space of the micellar template [17]. This type of interaction during mesophase formation was useful in the synthesis of mesostructured silica via an anionic-surfactant templating route in the presence of aminopropylsiloxane as a co-structure-directing agent [36]. On the other hand, the NR/WMS-NH_2_ series showed a decrease in *D*_p_ from 2.63 nm to 2.48 nm when increasing the APS loading level from 5 mol% to 15 mol%. The expansion of the template micelle might be traded off with a hydrophobic interaction between the propyl moieties of the organo-functional groups and the rubber molecules. This explanation was supported by a degree of *V*_p_ reduction at the same 10 mol% APS loading level, where the NR/WMS-0.10 (56%) exhibited a more reduced *V*_p_ than the WMS-0.10 (44%). Nevertheless, the introduction of NR into the mesopores should be limited by the mesostructured confined space due to its large molecular size. Our previous study demonstrated that the rubber phase was majorly entrapped in the silicate wall of the nanocomposite and highly dispersed throughout the mesostructured framework [13].

### 2.7. Electron Microscopy

**Figure 6:**
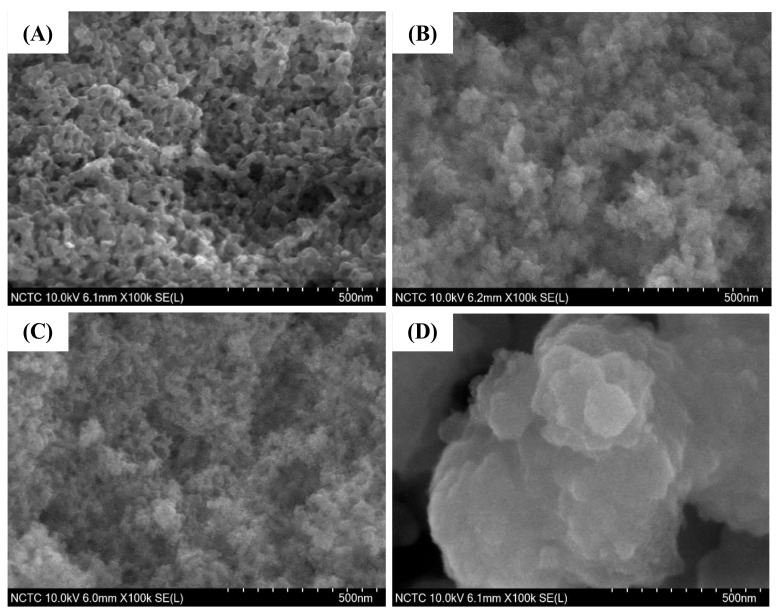
FE-SEM images of (**A**) WMS, (**B**) WMS-0.10, (**C**) NR/WMS and (**D**) NR/WMS-0.10 at magnification of 100,000×.

The FE-SEM images of pure silica WMS and NR/WMS nanocomposites prepared without and with functionalization are compared in Figure 6. The addition of rubber in the NR/WMS synthesis enhanced the agglomeration of silica particles when compared to the WMS. This observation was in accord with their N_2_ physisorption isotherms of which the pure silica WMS had a larger hysteresis loop at *P/P*_0_ > 0.8 than the NR/WMS (Figure 5A). From the particle size measurement using ImageJ software, the WMS and NR/WMS particles were 57–72 nm and 26–41 nm in size, respectively. The rubber phase, with its stiff nature, not only hampered the sol-gel reaction, resulting in a less condensed silicate framework, but also induced the particles’ packing [13]. The WMS-NH_2_ exhibited a larger particle size and more agglomerate than the WMS. A similar effect of surface functionalization was found for the NR/WMS nanocomposites. The particle size of the WMS-NH_2_ was 30–50 nm, while that of the NR/WMS-NH_2_ was 200–600 nm. The result confirmed the base-catalyzed hydrolysis and condensation of the silica precursor in the presence of aminopropylsilane. 

**Figure 7:**
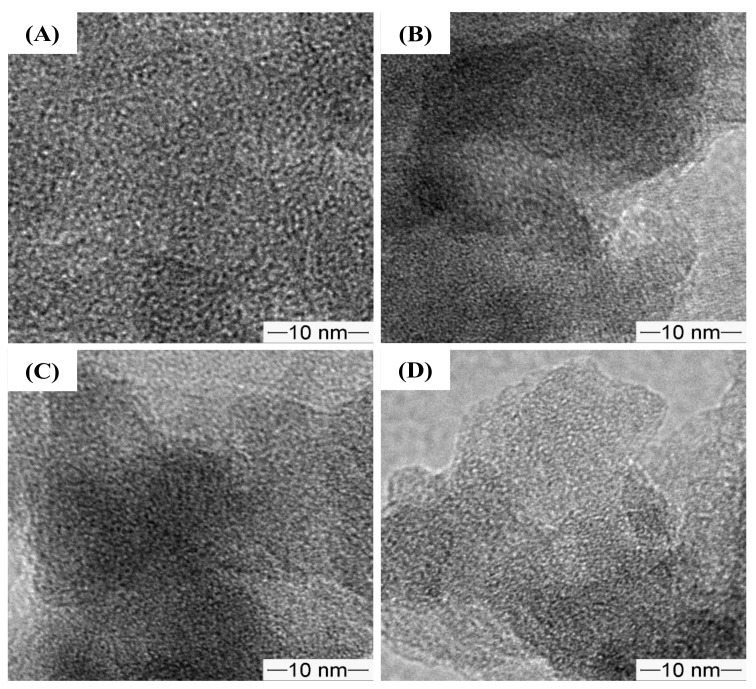
TEM images of (**A**) WMS, (**B**) WMS-0.10, (**C**) NR/WMS and (**D**) NR/WMS-0.10 at magnification of 100,000×.

As shown in Figure 7, the TEM images of the pristine and amino-functionalized NR/WMS nanocomposites revealed the uniform wormhole-like mesopores conventionally observed in WMS materials [13,16,33,37]. The result indicated that the mesostructured framework of the NR/WMS was preserved after the functionalization.

### 2.8. H_2_O Adsorption–Desorption Measurement

**Figure 8:**
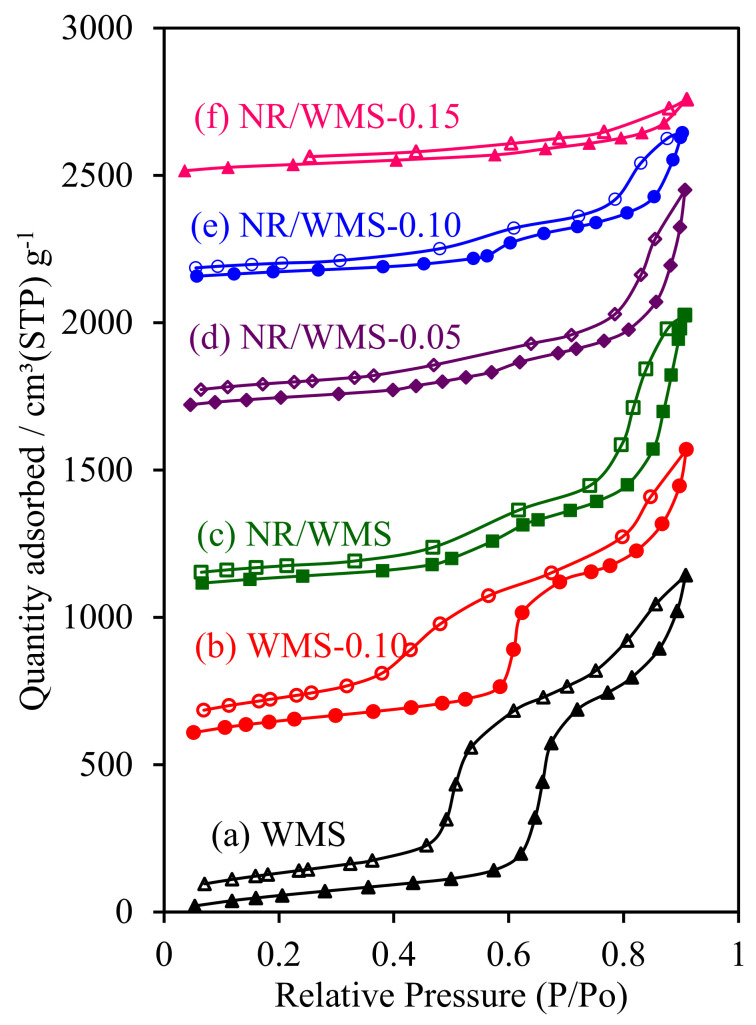
H_2_O adsorption–desorption isotherms of (a) WMS, (b) WMS-0.10, (c) NR/WMS, (d) NR/WMS-0.05, (e) NR/WMS-0.10 and (f) NR/WMS-0.15.

The H_2_O adsorption–desorption measurement was used to evaluate the effect of the aminopropyl group and/or NR on the hydrophobic properties of functionalized WMS and NR/WMS materials. Both of the pristine WMS and NR/WMS nanocomposites exhibited type IV isotherms with a hysteresis loop (Figure 8). The presence of rubber incorporated into the mesostructured silica reduced the size of the hysteresis loop, indicating that H_2_O desorbed from the surface of the NR/WMS more easily than that of the WMS. The pure silica WMS and NR/WMS nanocomposites had a *V*_mH2O_ of 70.4 and 43.7 cm^3^ g^−1^, respectively (Table 2). A decrease in the H_2_O affinity observed for the NR/WMS was explained by not only the hydrophobicity of the rubber phase but also the relatively small amount of silanol groups, compared to the WMS, as evidenced by FT-IR (Figure 1) analysis. 

The surface functionalization not only narrowed the hysteresis loops further, but also decreased the H_2_O adsorbed volume of the resulting WMS-NH_2_ and NR/WMS-NH_2_. With the increasing APS loading level in the nanocomposites, the monolayer adsorbed volume of H_2_O was decreased in the following order: NR/WMS (43.7 cm^3^ g^−1^) < NR/WMS-0.05 (42.1 cm^3^ g^−1^) < NR/WMS-0.10 (36.2 cm^3^ g^−1^) < NR/WMS-0.15 (33.2 cm^3^ g^−1^). The result was correlated with the reduced amount of exposed silanol groups in both functionalized materials via the surface grafting of an aminopropyl group (Figure 1). Although the ^29^Si CP/MAS-NMR result indicated that the WMS-0.10 had a higher degree of amino-functionalization (Table 1) and a smaller content of surface silanol groups than the NR/WMS-0.10 (Table 2), the former exhibited a higher H_2_O affinity than the latter. The result revealed that the hydrophobic environment created by the entrapped rubber was a major influence on the overall hydrophobicity of the NR/WMS-NH_2_ nanocomposites.

### 2.9. Adsorption Experiment

#### 2.9.1. Adsorption Kinetics

**Figure 9:**
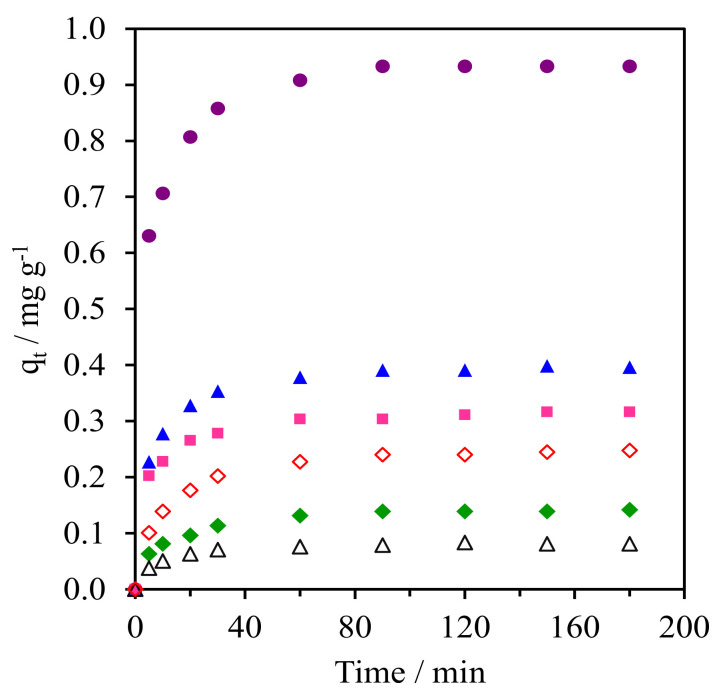
Kinetic curves of CFA adsorption onto (Δ) WMS, (◊) WMS-0.10, (⧫) NR/WMS, (●) NR/WMS-0.05, (▲) WMS-0.10 and (■) NR/WMS-0.15.

Figure 9 and Figure S2 (SM) shows the kinetics of CFA adsorption onto the pure silica WMS and NR/WMS nanocomposites prepared without and with an aminopropyl group. From the plot of the CFA adsorption capacity (*q*_t_) over time, all materials exhibited a fast CFA adsorption in the first 30 min, and then reached equilibrium within 60 min. The calculated parameters obtained from the different kinetic models and the corresponding correlation coefficients (R^2^) for each adsorbent are summarized in Table 3. Regardless of the adsorbent type and aminopropyl group content, the adsorption of CFA was well represented by pseudo-second order kinetics, which indicated that the adsorption phenomena involved chemical processes [3,38]. The WMS-NH_2_ (0.25 mg g^−1^) had a higher adsorption capacity than the pristine WMS (0.08 mg g^−1^). This result was explained by the combined electrostatic and hydrogen-bonding interaction between the amino groups on the functionalized silica surface and the carboxyl groups of the CFA molecule. 

The beneficial effect of the rubber phase was seen when compared with the adsorption capacity of the NR/WMS with that of the WMS. The NR/WMS showed a 1.8-fold higher capacity in CFA adsorption than the WMS, although the nanocomposite had inferior textural properties to the pure silica one. This indicates that the hydrophobicity of the NR played an important role in the adsorption onto the NR/WMS material. Nevertheless, if one compares the adsorption kinetics between WMS-0.10 and NR/WMS with similar *S*_BET_, it can be rationalized that the CFA adsorption preferentially occurred on the amino group. Bui et al. demonstrated that the adsorption of gemfibrozil onto SBA-15 can be enhanced by grafting hydrophobic organo-functional groups onto the adsorbent surface [38]. Accordingly, we believe that the hydrophobic rubber phase promoted the diffusion of the CFA molecules and retarded the competitive adsorption of H_2_O in the solution onto the NR/WMS surface, while the silanol groups were the main active centers responsible for the CFA adsorption via a hydrogen-bonding interaction [39]. The combination of NR and aminopropyl group enhanced the kinetics of the CFA adsorption onto the NR/WMS-*x* series. At the same amount of APS loading, the adsorption capacity of the NR/WMS-0.10 was around 1.6-fold larger than that of the WMS-0.10. The NR/WMS-0.05 had the highest adsorption capacity of 0.93 mg g^−1^, but the amount of CFA adsorbed was gradually decreased when the APS loading level was increased to 10 and 15 mol%. The lower adsorption capacity of the NR/WMS-0.10 and NR/WMS-0.15 was attributed to their poor textural properties (Table 2).

#### 2.9.2. Adsorption Isotherms

**Figure 10:**
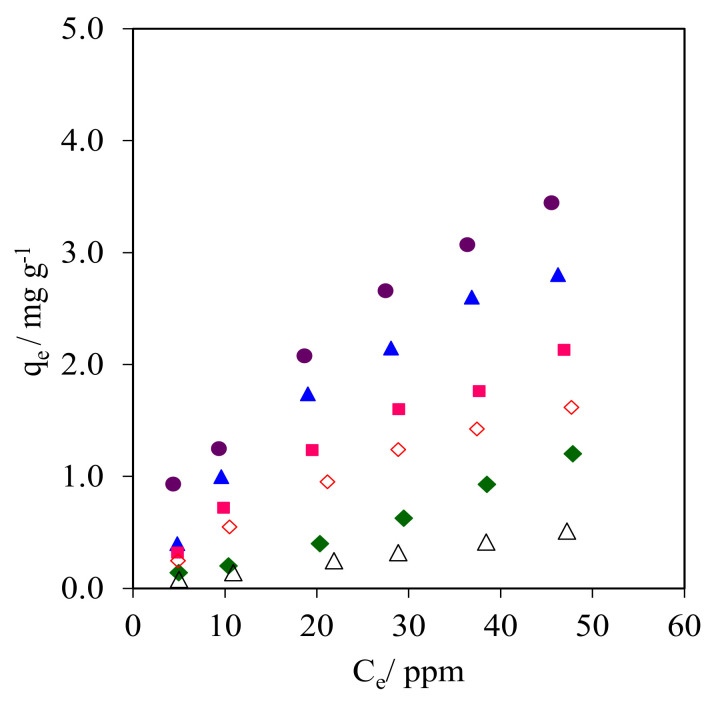
Isotherms of CFA adsorption onto (Δ) WMS, (◊) WMS-0.10, (⧫) NR/WMS, (●) NR/WMS-0.05, (▲) WMS-0.10 and (■) NR/WMS-0.15.

The isotherm plots obtained from the CFA adsorption onto the WMS and NR/WMS materials are compared in Figure 10 and Figure S3. The data were analyzed with the Langmuir and Freundlich mathematical expressions to postulate the model of CFA adsorption onto the pristine and functionalized materials. The CFA adsorption capacity observed for these adsorbents was ranked in the following descending order: NR/WMS-0.05 > NR/WMS-0.10 > NR/WMS-0.15 > WMS-0.10 > NR/WMS > WMS. The calculated parameters and R^2^ for each adsorbent are shown in Table 4. The isotherms of CFA adsorption on the amino-functionalized WMS and NR/WMS materials properly fitted with Langmuir models, while the Freundlich model was more suitable for the adsorption over the pristine WMS and NR/WMS materials as judged by the R^2^ values and data modeling approaches (Figure S3). The previous study revealed that the adsorption of CFA onto the amino-functionalized mesoporous silica materials was best described with the Langmuir adsorption model [3,17]. In our case, the isotherm study suggested that the CFA adsorption onto the surface of pure silica WMS and NR/WMS nanocomposites without and with the aminopropyl groups proceeded via a combination of different interaction forces [3,18,38]. The silanol groups mainly provided the hydrogen-bonding interaction, while the amino-functional groups offered both a hydrogen-bonding interaction and electrostatic force with the CFA molecules. According to Barczak, the amine-functionalized SBA-15 enhanced the adsorption capacity of pharmaceutical residue molecules in aqueous solution due to both a hydrogen-bonding interaction and electrostatic force [40]. The introduction of a rubber phase into the mesostructured silica inserted a weak hydrophobic interaction via the aromatic moieties of the CFA molecules. Lotfi et al. reported that the polyamidoamine/silica (PAMAM/SiO_2_) adsorbent exhibited a higher adsorption capacity of CFA compared to pure SiO_2_ adsorbent due to its hydrophobicity and amine-functionalized group [41]. Therefore, the presence of both the NR and aminopropyl groups in the NR/WMS-NH_2_ materials promoted not only the adsorption kinetics by reducing the competitive adsorption of H_2_O, but also molecular interactions with the CFA molecules, resulting in the enhanced adsorption performance.

#### 2.9.3. Adsorption Thermodynamics

The thermodynamic parameters of CFA adsorption were studied for the most effective adsorbent (NR/WMS−0.05) at different temperatures (298–318 K). The standard enthalpy change (Δ*S*°), standard entropy change (Δ*H*°), and Gibbs free energy (Δ*G*°) of adsorption were determined using the thermodynamic equation [42] presented in Equation (1):

(1) $$\ln\;\left(\frac{q_{e}}{C_{e}}\right)=-\frac{\Delta G{}^{\circ}}{RT}=\frac{\Delta S{}^{\circ}}{R}-\frac{\Delta H{}^{\circ}}{RT}\;$$

where *R* is the gas constant (8.314 J mol^−1^ K^−1^) and T is the absolute temperature (K). The value of ΔH° and ΔS° were, respectively, calculated from the slope and intercept of ln$\left(\frac{q_{e}}{C_{e}}\right)$versus 1/*T*. The obtained thermodynamics parameters (Δ*G*°, Δ*S*° and Δ*H*°) are summarized in Table 5. The value of Δ*G*° was negative, indicating the spontaneous nature of the CFA adsorption on the NR/WMS-NH_2_ materials. Moreover, the Δ*G*° values of the adsorption were increased with increasing temperature from 298 to 318 K, which suggests that the adsorption is more favorable at high temperatures [3]. The positive values of Δ*H*° and Δ*S*° suggest an endothermic process in which the randomness at the solid–solution interface increased during the adsorption due to adsorption and subsequent desorption [42,43].

#### 2.9.4. Reusability of NR/WMS-NH_2_

**Figure 11:**
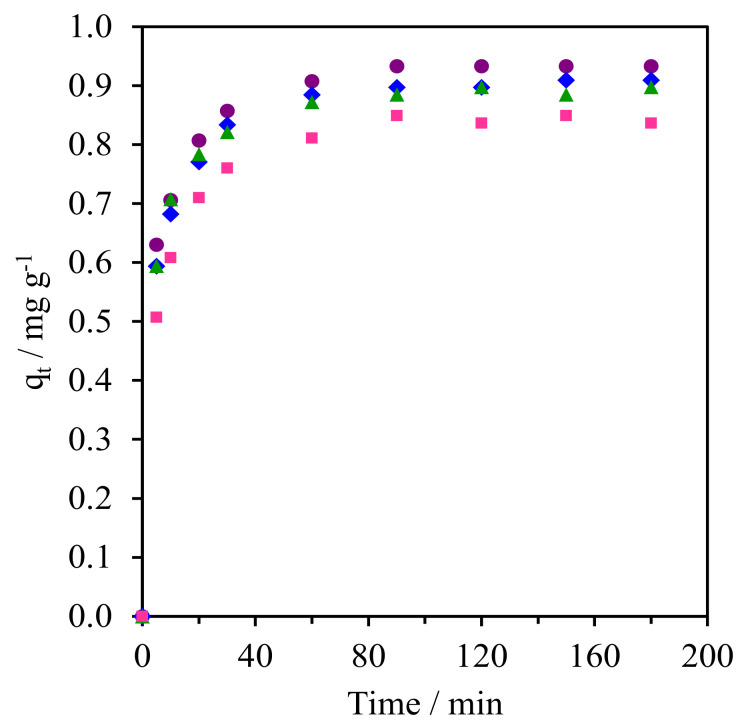
Reusability test of NR/WMS−0.05 in the CFA removal from aqueous solution. (●) Fresh, (▲) 1st Cycle, (⧫) 2nd Cycle and (■) 3rd Cycle.

The reusability test of NR/WMS-0.05 as the suitable adsorbent was carried out using the CFA concentration of 5 mg L^−1^ and the adsorbent to aqueous solution ratio was fixed at 1 g L^−1^. The spent adsorbent was separated from the mixture by filtration, followed by thoroughly washing with ethanol and drying at 90 °C for 12 h. As shown in Figure 11, the adsorbent could be reused at least three times, during which the adsorption capacity was slightly decreased from 0.93 to 0.84 mg g^−1^. The physicochemical properties of the adsorbent spent after the third cycle was characterized using XRD, TGA, and CHNS analyses. The XRD pattern of the reused adsorbent still showed a single reflection at 2*θ* around 1.5°–3.0°, although its intensity slightly decreased (Figure S4), indicating that the mesostructure of the NR/WMS-0.05 was not significantly changed by the repetitive uses and regeneration process. Similarly, the TGA analysis revealed no difference in the temperature-dependent weight loss behavior of the fresh and reused adsorbents (Figure S5). This implied that the rubber phase was retained in the mesostructured framework of the NR/WMS nanocomposite. However, the amount of nitrogen dropped around 16% (Table S1). These results indicate that the leaching of anchored amine groups from the adsorbent surface during their repetitive use in the adsorption was a major cause of their decreased capacity for CFA removal. Nevertheless, since the reused material still has a well-ordered mesostructured framework, it is possible to reactivate the spent adsorbent by grafting the APS to compensate for the amount of lost amine groups.

### 2.10. Comparison of Adsorbent Performance

The performance of different adsorbents reported so far for the CFA adsorption in aqueous solution are compared in Table 6. The pure silica SBA-15 (entry 1) exhibited the lowest adsorption capacity (0.07 mg g^−1^) due to its hydrophilic surface and lack of specific active sites for CFA adsorption. The functionalization of mesoporous silica with aminopropyl groups (entry 2) enhanced the capacity of the CFA adsorption via electrostatic and hydrogen-bonding interactions between the protonated amine groups (pK_b_ ≈ 3.4) and the negatively charged carboxyl groups of the CFA molecules (pK_a_ = 2.9) at neutral pH (~7) [3,17,36]. The coconut shell-derived activated carbon (entry 3) provided higher CFA adsorption capacity than the amine-functionalized mesoporous silica (entry 2), which is owed to not only its high surface area and porosity but also the hydrophobicity of its porous surface. The enhanced affinity for CFA molecules by hydrophobic carbonaceous materials originates from π→π and Van der Waals interactions [44]. The pillared bentonite modified with a cationic surfactant (entry 4) exhibited a high capacity for CFA adsorption due to the improved hydrophobic characteristics provided by the aliphatic chain of surfactant molecules [45]. In the case of NR/WMS-NH_2_ (entry 5), the presence of amine groups and rubber moieties as hydrophobicity improvers resulted in the highest adsorption performance (7.62 mg g^−1^). Moreover, the mesostructured framework, with the large mesopore volume and surface area of the adsorbents, encourages the intraparticle diffusion of adsorbate molecules [17,33].

## 3. Experimental Procedure

### 3.1. Material and Chemical Reagents

Tetraethyl orthosilicate (TEOS, AR grade, 99%), dodecylamine (DDA, AR grade, 98%), APS (AR grade, 97%), sulfuric acid (H_2_SO_4_) (AR grade, >95%) and clofibric acid (AR grade, >98.5%) were purchased from Sigma-Aldrich. THF (AR, grade, 99.5%) was a product of QRëC. Absolute ethyl alcohol (C_2_H_5_OH) (AR grade, 99.5%) was obtained from Macron Fine Chemicals. The commercial NR (STR-5L) was supplied by Thai Hua Chumporn Natural Rubber Co., Ltd. (Bangkok, Thailand). All chemical reagents were used without further purification.

### 3.2. Synthesis of Pure Silica WMS and WMS-NH_2_ Materials

The synthesis of the pure silica WMS and WMS*-*NH_2_ nanocomposites using DDA as an organic template and TEOS as the silica source was carried out as previously reported [17]. The pure silica WMS was synthesized via a sol-gel procedure using DDA as an organic template and TEOS as the silica source, with a gel molar composition of 0.05 TEOS: 0.02 DDA: 2.94 H_2_O: 0.74 THF. Typically, 3.75 g of DDA was dissolved in 53.05 g of deionized water and 13.34 g of THF under stirring to obtain a template solution. To this solution, 10.50 g of TEOS was added dropwise at room temperature under stirring for 24 h. The mixture was aged in an oven at 40 °C for 24 h. The resulting white solid was achieved by filtration, thoroughly washing with ethanol, and drying in an oven at 60 °C for 24 h. The template removal was carried out by extraction with a 0.05 M sulfuric acid/ethanol solution at 80 °C for 4 h, followed by drying in an oven at 60 °C for 24 h. In the case of the WMS-NH_2_ material, the TEOS and APS were mixed in THF prior to the dropwise addition into the template solution. A molar ratio of APS/(APS+TEOS) was maintained at 0.10. Subsequently, the resulting mixture was stirred at room temperature for 24 h, and then aged in an oven at 40 °C for another 24 h. The recovery of the white solid and the removal of the organic template were carried out by the same procedure as the synthesis of pure silica WMS. The WMS-NH_2_ obtained was designated as WMS−0.10.

### 3.3. Synthesis of NR/WMS and NR/WMS-NH_2_ Nanocomposites 

A series of NR/WMS-NH_2_ with different amine contents were synthesized via the in-situ sol-gel method adapted from the procedure described by Nuntang et al. [13]. Firstly, 0.50 g of a NR sheet was swollen in TEOS at room temperature for 16 h, and then dissolved in THF (13.34 g) under agitation overnight. Next, 3.75 g of DDA was added into the resulting homogeneous mixture, followed by the dropwise addition of a solution containing TEOS, APS and THF. After stirring for 0.5 h, deionized water (53.05 g) was dropped into the mixture under stirring. The total amount of TEOS used was 10.5 g, while the molar ratio of APS/(APS+TEOS) varied from 0.05 to 0.15. Subsequently, the mixture was allowed to stand at room temperature for 24 h, and then aged in an oven at 40 °C for 3 d. The white solid was precipitated in 50 mL of ethanol, filtered, thoroughly washed with ethanol, and dried in an oven at 60 °C for 24 h. Finally, the organic template was removed by the same procedure as described in Section 2.2. The resulting amine-functionalized nanocomposites were designated as NR/WMS−*x*, where *x* represents the APS/(APS+TEOS) molar ratio used in the synthesis. A non-functionalized NR/WMS nanocomposite was also synthesized using a similar synthesis procedure and composition as the NR/WMS-NH_2_ series, but without adding APS.

### 3.4. Characterization of Adsorbents

A thermogravimetric analysis (TGA) was used to obtain the thermal decomposition behavior of the materials. The analysis was carried out on a PerkinElmer PyrisDiamond under a dry air flow of 50 mL min^−1^ with a heating rate of 10 °C min^−1^ from 40 °C to 1000 °C. The element analysis was performed on a LECO Corporation 628 Series CHNS/O elemental analyzer to determine the total amount of nitrogen incorporated into the materials.

Fourier-transform infrared spectroscopy (FTIR) was applied to verify the presence of rubber and amine-functional groups in the nanocomposites. The FTIR spectra were collected over the wavenumber of 4000–400 cm^−1^ at room temperature in attenuated total reflectance (ATR) mode on a PerkinElmer Spectrum One Fourier-transform infrared spectrometer. 

The type and relative concentration of silica species presenting in the materials were measured by solid-state ^29^Si cross polarization/magic angle spinning nuclear magnetic resonance spectroscopy (^29^Si CP/MAS NMR). The NMR spectra were acquired on a Bruker Ascend 400 WB Fourier-transform nuclear magnetic resonance spectrometer. The instrument was operated at 79.4 MHz with a sample spinning frequency of 5 kHz, a delay time of 3 s and a CP contact time of 2.5 ms. Sodium trimethylsilylpropanesulfonate was used as an internal standard to quote the chemical shifts of ^29^Si MAS NMR spectra in parts per million (ppm). The relative band area of each silica species was achieved by a curve-fitting analysis using a series of Gaussian curves (OriginPro 8.5 software).

The mesostructured arrangement of the synthesized materials was analyzed by power X-ray diffraction (XRD) using a Bruker D8 ADVANCE diffractometer equipped with Cu Kα radiation at 40 kV and 40 mA. The diffraction was measured in the range of 2*θ* = 0.5–10° with a scanning step of 0.02° and a count time of 1 s. The repeating distance (*a*_0_) between the pore centers of the mesostructure was calculated from the interplanar spacing of (100) plane (*d*_100_) using the formula; *a*_0_ = 2*d*_100_/$\sqrt{3}$. 

The N_2_ physisorption measurement was used to access the textural properties of the materials without and with the amine-functional group. The adsorption-desorption of N_2_ was carried out at −196 °C using a surface area and porosity analyzer (Micromeritics ASAP 2020). Prior to the measurement, the sample was degassed at 150 °C for 2 h. The specific surface area (*S*_BET_) was calculated using the Brunauer−Emmett−Teller (BET) method with the adsorption data in the relative pressure (*P/P*_0_) range of 0.02–0.2, while the total pore volume (*V*_t_) was determined from the accumulative volume of N_2_ adsorbed at a *P/P*_0_ of about 0.990. The external surface area (*S*_ext_) and primary mesopore volume (*V*_p_) were determined using the *t*-plot method. The pore size (*D*_p_) was determined by the Barrett–Joyner–Halenda (BJH) method using the adsorption branch data of N_2_ sorption isotherms. 

The effect of functionalization on the morphology of the resulting materials was investigated by field-emission scanning electron microscopy (FE-SEM). The FE-SEM images were recorded on a Hitachi SU5000 instrument at an acceleration voltage of 40 kV. The sample powder was deposited on carbon tape, followed by platinum coating. The particle size and interparticle voids were measured using ImageJ software. Transmission electron microscopy (TEM) was used to confirm the mesoporous structure of the nanocomposites. The TEM micrographs were observed at a magnification of 150,000× using a JEOL 2010 transmission electron microscope operated at 200 kV.

The materials’ hydrophobicity was evaluated by H_2_O adsorption–desorption measurement using a BEL Japan BELSORP-max instrument. Typically, a sample powder was pretreated under vacuum at 150 °C for 2 h. The isotherm measurement was conducted at 25 °C. The monolayer adsorbed volume (*V*_m_) of H_2_O was calculated from the analysis of adsorption data in the *P/P*_0_ range below 0.2.

### 3.5. Adsorptive Removal of CFA

#### 3.5.1. Adsorption Kinetics

The performance of the WMS and NR/WMS without and with the amine-functional group in the adsorptive removal of CFA in aqueous phase was evaluated by a shake-flask method. Before being used, the adsorbents were pretreated at 80 °C for 4 h. Adsorption assays were carried out at 35 °C as a batch experiment in which the CFA was dissolved in deionized water to obtain an initial concentration of 5 mg L^−1^, and the ratio of adsorbent to aqueous solution was maintained at 1 g L^−1^. The mixture was agitated (150 rpm) in an orbital shaker. The concentration of CFA remaining at different time intervals was measured by UV spectrophotometry using a Biochrom Libra S22 UV-VIS spectrophotometer. 

The experimental data were fitted with the pseudo-first, pseudo- and Ritchie-second-order kinetic models to obtain the kinetic parameters. 

The linearized pseudo-first order rate expression [48] was defined as Equation (2);

(2) $$\log\left(q_{m}\;q_{t}\right)=\log\;\left(q_{m}\right)\;-\frac{k_{1}}{2.303}t$$

The linearized pseudo-second order rate expression [49] was defined as Equation (3);

(3) $$\frac{t}{q_{t}}=\;\frac{1}{k_{2}q_{e}^{2}}+\;\frac{t}{q_{e}}$$

where $q_{t}$ and $q_{e}$ are the amount of CFA adsorbed at any given time (t) and at equilibrium (mg g^−1^), respectively, and $k_{2}$ is the pseudo-second order rate constant (g mg^−1^ min^−1^) determined from the plots of $t/q_{t}$ versus t.

The linear form of the Ritchie-second-order equation [50] can be expressed as Equation (4);

(4) $$\frac{1}{q_{t}}=\frac{1}{k_{r}q_{e}t}+\frac{1}{q_{e}}$$

where $k_{r}$ is the Ritchie-second-order rate constant (min^−1^) derived from the plots of $1/q_{t}$ versus 1/t.

#### 3.5.2. Adsorption Isotherm

Adsorption isotherm assays were performed in a similar manner to the adsorption kinetic study, except that the initial concentration of CFA solution was varied from 10 to 50 mg L^−1^. The liquid samples were only taken at the equilibrium time, which was calculated in the adsorption kinetic experiment.

The adsorption capacity of the materials at the equilibrium concentration of CFA solution was plotted to access the adsorption behavior according to Langmuir and Freundlich isotherm models [51]. 

The Langmuir isotherm in a linear form was expressed as Equation (5);

(5) $$\frac{1}{q_{e}}=\frac{1}{q_{m}}+\frac{1}{k_{L}q_{m}C_{e}}$$

where $q_{m}$ is the maximum adsorption capacity (µg g^−1^) and $k_{L}$ is the Langmuir constant. 

Equation (6) shows the linearized Freundlich isotherm expression;

(6) $$\ln q_{e}=\ln k_{F}+\frac{1}{n}\ln C_{e}$$

where $k_{F}$ is the Freundlich constant and n is the adsorption intensity (dimensionless).

## 4. Conclusions

A series of ordered NR/WMS-NH_2_ with wormhole-like mesostructures were successfully synthesized as new hybrid/composite-type solid adsorbents by using an in situ sol-gel process with the direct co-condensation method. The incorporation of NR and/or an organo-functional group into the WMS structure decreased the amount of free silanol groups and improved the hydrophobicity of the materials. The ordering structure and the textural properties were decreased with increasing APS loading levels. The NR/WMS-NH_2_ with 5 mol% APS loading had the maximum CFA adsorption capacity of 6.29 mg g^−1^, showing it as a promising application for the removal of CFA molecules dissolved in water. It was found that the pseudo-second order model fitted the sorption kinetic data better than the Ritchie-second order kinetic model. However, the CFA adsorption capacity of the NR/WMS-NH_2_ decreased as the APS loading level increased over 5 mol% due to its low surface area and pore volume, which led to a reduction in the sorption capacity of the adsorbent. The surface properties of the adsorbent had a strong influence on the CFA adsorption mechanisms though hydrophobic interaction, electrostatic interaction, and also possibly via hydrogen bonding.

## Figures and Tables

**Table 1:** Relative intensities of the T^m^ and Q^n^ species obtained from the deconvolution of ^29^Si CP/MAS NMR spectra.

Sample	Si Species Distribution (%)					ΣT^m^/Σ(T^m^ + Q^n^) (%)
	T^2^	T^3^	Q^2^	Q^3^	Q^4^	
WMS	–	–	11.89	68.79	19.32	–
WMS-0.10	4.78	12.75	8.54	49.38	24.55	17.53
NR/WMS	–	–	17.56	70.43	12.01	–
NR/WMS-0.10	3.12	11.97	8.93	52.31	23.67	15.09

**Table 2:** Physicochemical properties of pure silica WMS, NR/WMS and NR/WMS-NH_2_ materials synthesized under various conditions.

Sample ^a^	Amine Concentration (mmol_N_ g^−1^)		*S*_BET_ ^c^ (m^2^ g^−1^)	*S*_ext_ ^d^ (m^2^ g^−1^)	*D*_p_ ^e^ (nm)	*V*_t_ ^f^ (cm^3^ g^−1^)	*V*_p_ ^g^ (cm^3^ g^−1^)	*d*_100_ ^h^ (nm)	*a*_0_ ^i^ (nm)	*W*_t_ ^j^ (nm)	*V*_m_ ^k^ (cm^3^ g^−1^)
	Theoretical	Experimental ^b^									
WMS	0.00	0.00	986	469	2.80	2.41	0.52	4.51	5.21	2.41	70.4
WMS-0.10	1.54	1.47	420	102	2.94	1.05	0.29	4.70	5.43	2.49	58.1
NR/WMS	0.00	0.00	492	241	2.90	1.34	0.23	4.91	5.67	2.77	43.7
NR/WMS-0.05	0.81	0.43	378	184	2.63	1.06	0.15	4.80	5.55	2.92	42.1
NR/WMS-0.10	1.54	1.23	238	110	2.55	0.92	0.10	4.75	5.49	2.94	36.2
NR/WMS-0.15	2.20	1.84	115	21	2.48	0.14	0.08	4.73	5.46	2.98	33.2

^a^ Extracted samples, ^b^ Determined by CHNS/O analyzer, ^c^ BET surface area, ^d^ External surface area determined from *t*-plot curves, ^e^ Pore diameter calculated using the BJH method, ^f^ Total pore volume, ^g^ Mesopore volume, ^h^ Interplanar spacing of (100) plane (*d*_100_) obtained from XRD analysis, ^i^ The repeat distance (*a*_0_) between the pore centers of the mesostructure was calculated from *a*_0_ = 2*d*_100_/3^½^, ^j^ The framework wall thickness was determined by subtracting the BJH mesopore size from the repeat distance between the pore centers, ^k^ Monolayer adsorbed volume of H_2_O.

**Table 3:** Kinetic parameters of CFA adsorption onto the adsorbent.

Adsorbent	*q_e_*_,exp_ (mg g^−1^)	Pseudo-First Order			Pseudo-Second Order			Ritchie-Second Order		
		*k*_1_ (min^−1^)	*q*_e,cal_ (mg g^−1^)	R^2^	*k*_2_ (g mg^−1^ min^−1^)	*q*_e,cal_ (mg g^−1^)	R^2^	*k*_r_ (L min^−1^)	*q_e_*_,cal_ (mg g^−1^)	R^2^
WMS	0.08	0.07	0.07	0.981	1.84	0.08	1.000	0.17	0.08	0.997
WMS-0.10	0.25	0.06	0.20	0.974	0.46	0.26	0.999	0.14	0.23	0.996
NR/WMS	0.14	0.05	0.11	0.977	0.79	0.15	0.995	0.18	0.13	0.968
NR/WMS-0.05	0.93	0.07	0.30	0.986	0.33	0.95	1.000	0.42	0.92	0.959
NR/WMS-0.10	0.40	0.05	0.18	0.981	0.56	0.41	1.000	0.27	0.39	0.991
NR/WMS-0.15	0.32	0.05	0.13	0.987	0.82	0.32	0.999	0.38	0.30	0.953

**Table 4:** Isotherm parameters of CFA adsorption onto the adsorbent.

Adsorbent	Langmuir			Freundlich		
	*k*_L_ (L mg^−1^)	*q*_m_ (mg g^−1^)	*R* ^2^	*n*	*k*_F_	*R* ^2^
WMS	0.008	1.81	0.996	1.13	0.017	0.999
WMS-0.10	0.016	3.73	0.993	1.38	0.103	0.976
NR/WMS	0.006	3.13	0.969	0.84	0.012	0.999
NR/WMS-0.05	0.026	6.29	0.999	1.55	0.305	0.993
NR/WMS-0.10	0.022	5.66	0.998	1.50	0.230	0.993
NR/WMS-0.15	0.020	4.30	0.997	1.44	0.149	0.991

**Table 5:** Thermodynamic parameters for CFA adsorption onto NR/WMS-0.05 at different temperatures.

Temperature (K)	Δ*G*° (kJ mol^−1^)	Δ*H*° (kJ mol^−1^)	Δ*S*° (kJ mol^−1^ K^−1^)
298	−13.18	8.08	71.36
308	−13.90		
318	−14.61		

**Table 6:** Maximum capacities (q_m_) of CFA adsorption onto different adsorbents.

Entry	Adsorbent	q_m_, (mg g^−1^)	Reference
1	SBA-15	0.07	[46]
2	Amine-functionalized mesoporous silica	0.26	[3]
3	Activated carbon from coconut shell	2.48	[47]
4	Metal modified inorganic-organic pillared clays	4.63	[45]
5	NR/WMS-NH_2_	6.29	This study

## Data Availability

The data presented in this study are available in the article and the Supplementary Data.

## Supplementary Materials

The following supporting information can be downloaded at: https://www.mdpi.com/article/10.3390/molecules28052330/s1, Figure S1: FTIR spectra of (a) WMS, (b) WMS-0.10, (c) NR/WMS, (d) NR/WMS-0.05, (e) NR/WMS-0.10, and (f) NR/WMS-0.15, Figure S2: Kinetic curves of CFA adsorption onto (a) WMS, (b) WMS-0.10, (c) NR/WMS, (d) NR/WMS-0.05, (e) NR/WMS-0.10, and (f) NR/WMS−0.15, Figure S3: Kinetic curves of CFA adsorption onto (a) WMS, (b) WMS-0.10, (c) NR/WMS, (d) NR/WMS-0.05, (e) NR/WMS-0.10, and (f) NR/WMS-0.15, Figure S4: XRD patterns of (a) NR/WMS-NH_2_ and (b) spent NR/WMS-NH_2_, Figure S5: Weight loss curves of (a) NR/WMS-NH2 and (b) spent NR/WMS-NH2, Table S1: Chemical composition of NR/WMS-NH2 and spent NR/WMS-NH2.

## Sample Availability

Samples of the compounds are not available from the authors.
