# Supplementary material for: Amine-Functionalized Natural Rubber/Mesostructured Silica Nanocomposites for Adsorptive Removal of Clofibric Acid in Aqueous Phase
Source: Molecules. 2023 Mar 2;28(5):2330. doi: 10.3390/molecules28052330 (PMC10004768; doi:10.3390/molecules28052330)
Supplement: Supplementary file 1 [file molecules-28-02330-s001.zip › molecules-2183989-supplementary.pdf]

Supplementary Materials

AMINE-FUNCTIONALIZED NATURAL RUBBER/MESOSTRUCTURED SILICA NANOCOMPOSITES FOR ADSORPTIVE REMOVAL OF CLOFIBRIC ACID IN AQUEOUS PHASE

Satit Yousatit,[1,2] Witsarut Rungruangwattanachot,[1] Natthakit Yuwawanitchakorn,[1] Sakdinun Nuntang,[3] Patiparn Punyapalakul,[4] Chawalit Ngamcharussrivichai[1,2,5,*]

[1] *Department of Chemical Technology, Faculty of Science, Chulalongkorn University, Pathumwan, Bangkok 10330, Thailand*

[2] *Center of Excellence in Catalysis for Bioenergy and Renewable Chemicals (CBRC), Faculty of Science, Chulalongkorn University, Pathumwan, Bangkok 10330, Thailand*

[3] *Industrial Chemistry Innovation Programme, Faculty of Science, Maejo University, Chiang Mai 50290, Thailand*

[4] *Research Unit Control of Emerging Micropollutants in Environment, Faculty of Engineering, Chulalongkorn University, Pathumwan, Bangkok 10330, Thailand*

[5] *Center of Excellence on Petrochemical and Materials Technology (PETROMAT), Chulalongkorn University, Pathumwan, Bangkok 10330, Thailand*

[*] Corresponding author. Tel.: +66 2218 7528; fax: +66 2255 5831.

E-mail address: Chawalit.Ng@Chula.ac.th

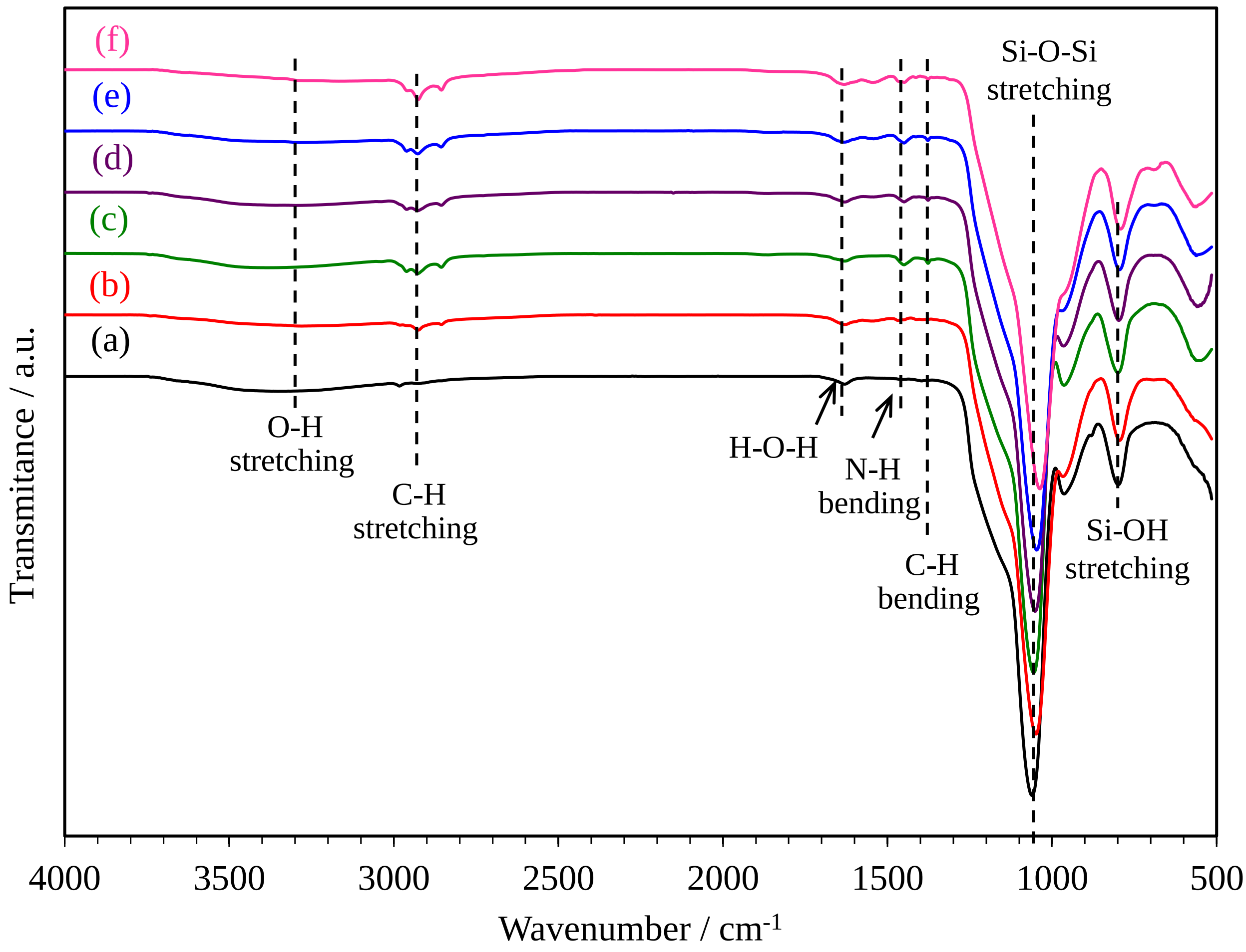

Figure S1. FTIR spectra of (a) WMS, (b) WMS-0.10, (c) NR/WMS, (d) NR/WMS-0.05, (e) NR/WMS-0.10 and (f) NR/WMS-0.15.

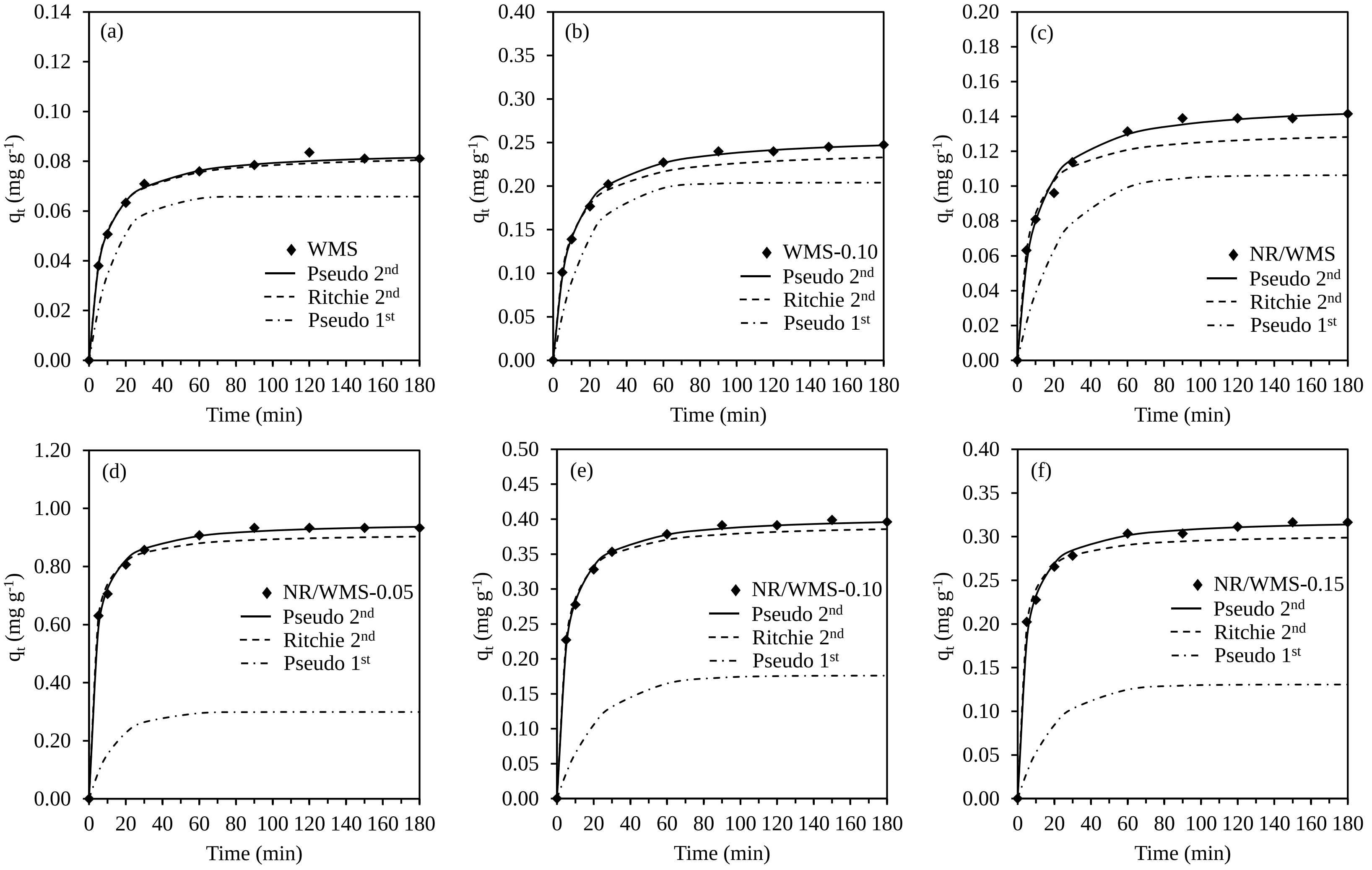

Figure S2. Kinetic curves of CFA adsorption onto (a) WMS, (b) WMS-0.10, (c) NR/WMS, (d) NR/WMS-0.05, (e) WMS-0.10, and (f) NR/WMS-0.15.

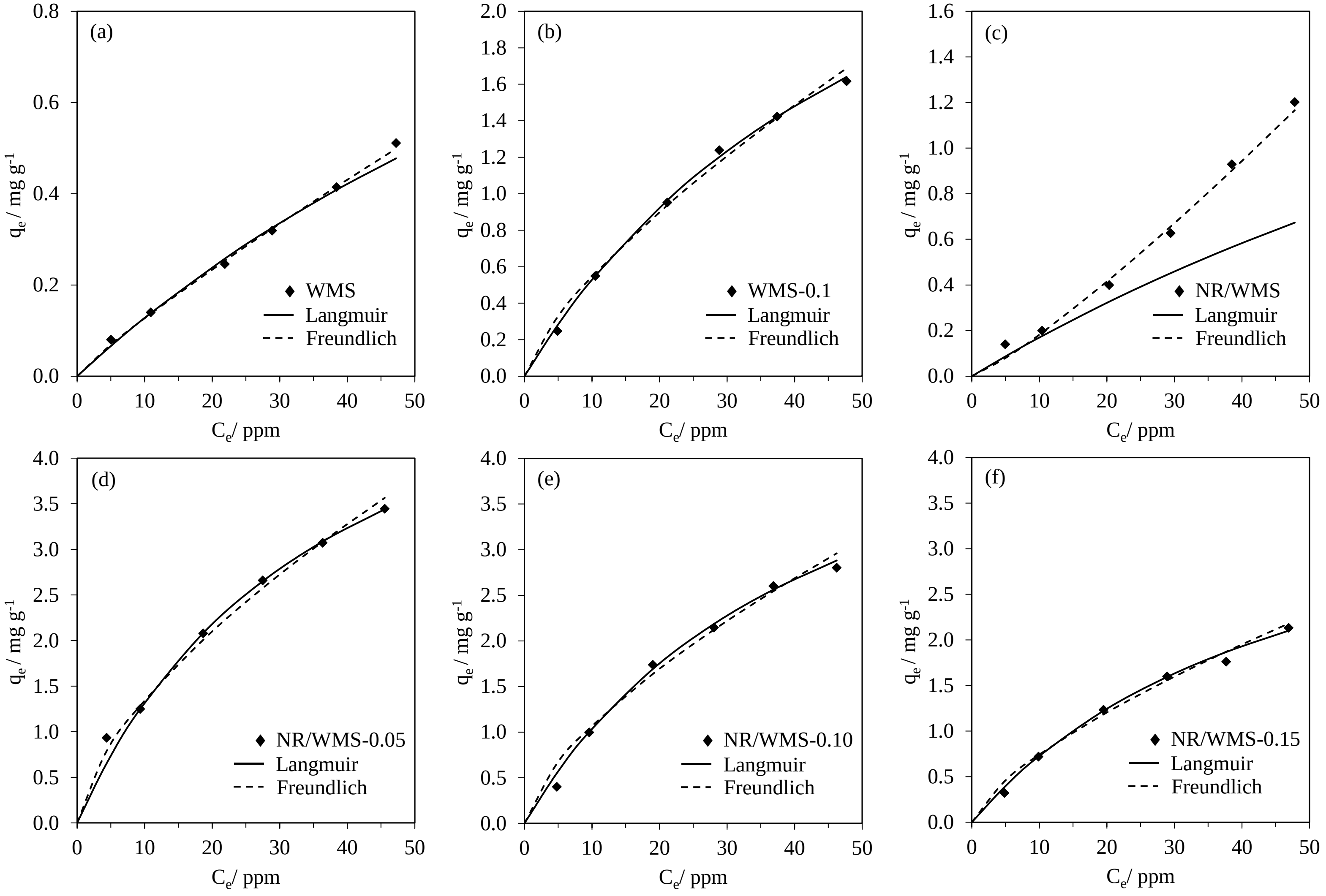

Figure S3. Kinetic curves of CFA adsorption onto (a) WMS, (b) WMS-0.10, (c) NR/WMS, (d) NR/WMS-0.05, (e) WMS-0.10, and (f) NR/WMS-0.15.

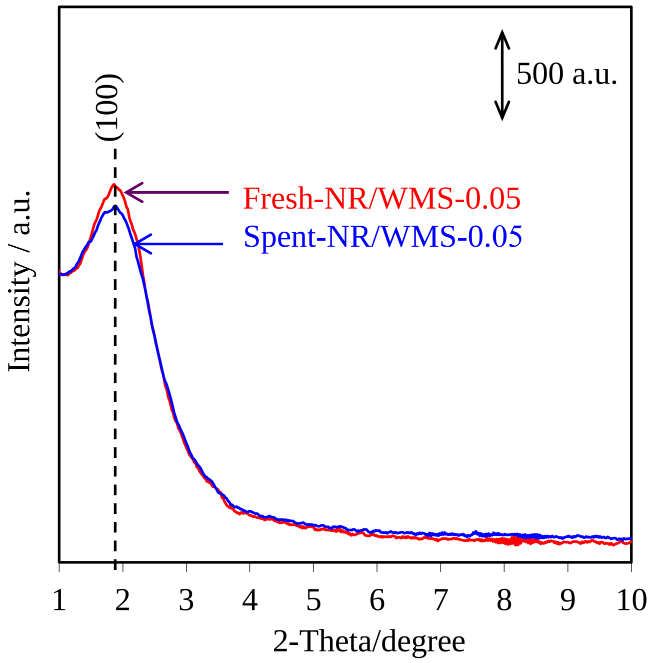

Figure S4. XRD patterns of (a) NR/WMS-NH_2 and (b) spent NR/WMS-NH_2.

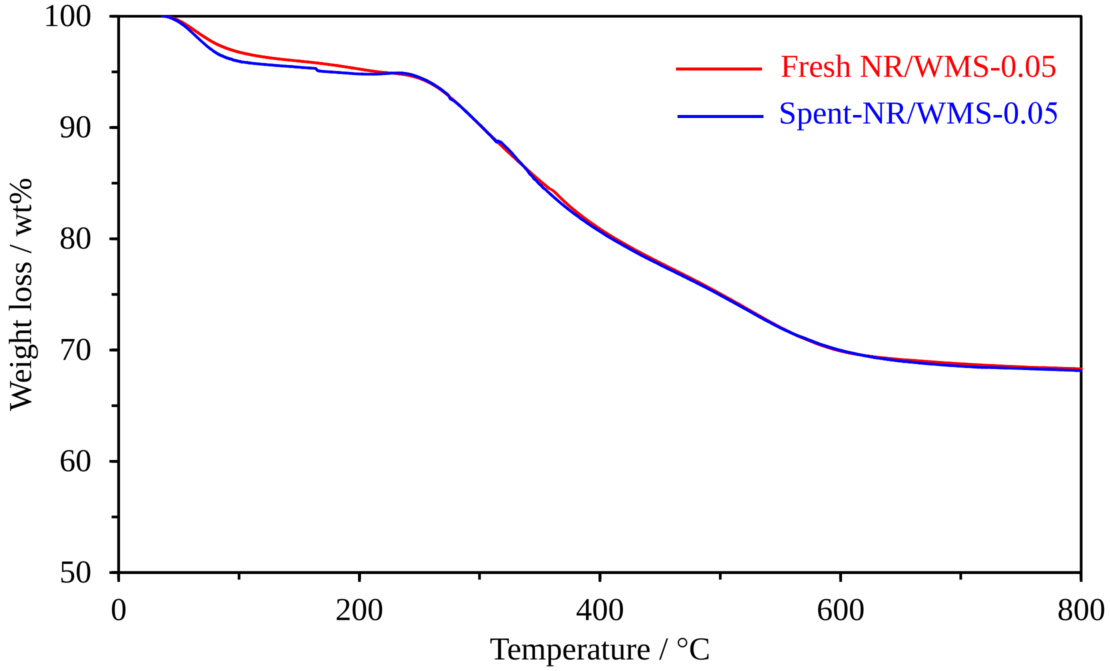

Figure S5. Weight loss curves of (a) NR/WMS-NH_2 and (b) spent NR/WMS-NH_2.

Table S1 Chemical composition of $NR/WMS-NH_2$ and spent $NR/WMS-NH_2$

Sample	Carbon content[a] (wt%)	Silica content[a] (wt%)	Nitrogen content[b] (mmol g^{-1})
NR/WMS-0.05	26.6	68.3	0.43
Spent- NR/WMS-0.05	26.7	68.1	0.36

n.d. = not determined.

[a] Determined by thermogravimetric analysis.

[b] Determined by CHNS analyzer.
